# Long-Term Follow-up Posthematopoietic Stem Cell Transplantation in a Japanese Patient with Type-VII Mucopolysaccharidosis

**DOI:** 10.3390/diagnostics10020105

**Published:** 2020-02-16

**Authors:** Kenji Orii, Yasuyuki Suzuki, Shunji Tomatsu, Tadao Orii, Toshiyuki Fukao

**Affiliations:** 1 Department of Pediatrics, Gifu University Graduate School of Medicine, Yanagido 1-1, Gifu 501-1194, Japan; stomatsu@nemours.org (S.T.);; 2 Medical Education Development Center, Gifu University, Yanagido 1-1, Gifu 501-1194, Japan; 3 Nemours/Alfred I. Dupont Hospital for Children, 1600 Rockland Rd., Wilmington, DE 19803, USA; 4 Department of Pediatrics, Thomas Jefferson University, Philadelphia, PA 19144, USA

**Keywords:** mucopolysaccharidoses, Sly syndrome, activity of daily living, hematopoietic stem cell transplantation

## Abstract

The effectiveness of hematopoietic stem cell transplantation (HSCT) for type-VII mucopolysaccharidosis (MPS VII, Sly syndrome) remains controversial, although recent studies have shown that it has a clinical impact. In 1998, Yamada et al. reported the first patient with MPS VII, who underwent HSCT at 12 years of age. Here, we report the results of a 22-year follow-up of that patient post-HSCT, who harbored the p.Ala619Val mutation associated with an attenuated phenotype. The purpose of this study was to evaluate changes in physical symptoms, the activity of daily living (ADL), and the intellectual status in the 34-year-old female MPS VII patient post-HSCT, and to prove the long-term effects of HSCT in MPS VII. Twenty-two years after HSCT, the β-glucuronidase activity in leukocytes remained at normal levels, and urinary glycosaminoglycan excretion was reduced and kept within normal levels. At present, she is capable of sustaining simple conversation, and her intellectual level is equivalent to that of a 6-year-old. She can walk alone and climb upstairs by holding onto a handrail, although she feels mild pain in the hip joint. The cervical vertebrae are fused with the occipital bone, causing dizziness and light-headedness when the neck is bent back. Overall, her clinical condition has been stabilized and kept well for long-term post-HSCT, indicating that HSCT is a therapeutic option for MPS VII.

## 1. Introduction

Mucopolysaccharidosis VII (MPS VII; Sly syndrome; OMIN#253220) is an autosomal recessive lysosomal storage disorder (LSD) that is caused by a deficiency of the activity of β-glucuronidase (GUSB: β-d-glucuronoside Enzyme Commission (EC) number: 3.2.1.31; GUSB: MIM611499)[1,2]. The deficiency of this enzyme leads to the accumulation of specific glycosaminoglycans (GAGs), i.e., chondroitin sulfate (CS), dermatan sulfate (DS), and heparan sulfate (HS). This disease was first clinically described by Sly et al. in a patient whose features were similar to those of the patient described here [1]. Although clinical phenotypes are widely seen from the most severe phenotype with hydrops fetalis to the attenuated phenotype without central nervous system (CNS) involvement, patients with MPS VII often show short stature, hepatosplenomegaly, progressive skeletal deformities of the thorax and spine, and frequent symptomatic pulmonary infections before 2 years of age. Intellectual disability also appears at 2 years of age. Montaño et al. reported that 34% of patients were diagnosed prenatally or below 1 year of age, 16% from 1 year to 3 years of age, 11% from 3 years to 5 years of age, 20% from 5 years to 10 years of age, and 12% over 10 years of age [3]. Thirteen patients with MPS VII who had the infantile form with a history of hydrops fetalis and survived childhood showed various clinical manifestations from mild to severe [3]. Radiologic changes of “dysostosis multiplex” involve the skull, spine, ribs, and long and short tubular bones. MPS VII patients with the most severe phenotype have hydrops fetalis at birth and often do not survive more than a few months. Patients with mild manifestations of MPS VII have survived into the fifth decade of life. Fifty-eight different pathogenic mutations in the *GUSB* gene have been identified to date [4]. A genotype/phenotype analysis indicated that the more severe phenotype was associated with truncating mutations.

At present, two therapeutic options are available: enzyme replacement therapy (ERT) and hematopoietic stem cell transplantation (HSCT). For ERT, a recombinant form of human GUS (vestronidase alpha) has been recently developed and used successfully [5], giving rise to a reduction of urinary GAGs and an improvement of the organomegaly [6]. However, the intravenously injected rhGUS does not cross the blood-brain-barrier and provides no effect on neurological signs, while HSCT can bring the enzyme into the brain via its secretion from donor-derived microglial cells and prevent or slow the neurological deterioration [7]. As learned from other MPS types, HSCT should be performed at an early stage of these diseases in the absence of pre-existing neurological damage. To date, several institutes have encountered HSCT in MPS VII patients. Although benefits in MPS VII treated with HSCT have been observed regarding the somatic features of the disease, the impact on cognitive function remains uncertain because of a lack of long-term observation of neurocognitive benefit.

We previously reported the first HSCT case with MPS VII with 2 years of follow-up post-HSCT after the patient underwent HSCT at 12 years of age [8]. Here we have followed up this patient over 20 years post-HSCT, and evaluated the clinical condition, including physical activity and neurocognitive function. 

## 2. Case Report

### 2.1. Diagnosis

Informed consent was obtained from her guardians. The study was approved by the ethics committee on human research at Gifu University (IRB number; 24-176, and 26-411) and followed the ethical principles of the Declaration of Helsinki. The patient was born at a gestational age of 38 weeks from an uncomplicated first pregnancy of related parents, weighing 2800 g (+0.28 standard deviation (SD)), birth length of 47.2 cm (−0.45 SD), and a head circumference of 33.4 cm (+0.29 SD). She had coarse facial features, hepatosplenomegaly, umbilical herniation, peripheral pulmonary stenosis, ventricular septal defect, severe neonatal jaundice, and abnormal peripheral leukocytes with striking coarse metachromatic granules. The diagnosis was established at the age of 1 month by the demonstration of a pronounced increase in the excretion of the urinary DS and CS and deficiency of β-glucuronidase in leukocytes. At 6 years of age, Tomatsu et al. identified the mutation in her β-glucuronidase cDNA producing the p.Ala619Val substitution [9]. She suffered from recurrent acute otitis media starting at several months of age, and mild deafness was first recognized at the age of 5 years. Developmental delay was noticed at 3 years of age, after which intellectual disability progressed gradually. Bone deformities (lumbar gibbus, bilateral femoral caput hypoplasia) worsened steadily. She became wheel-chair bound at 10 years of age because of gait disturbances, hip pain, and shortness of breath. Her grasp weakened until she could not lift her handbag. She suffered recurrent upper respiratory tract and middle ear infections. To attempt to reverse her progressive neurologic and somatic deterioration, the parents requested that a HSCT be considered [9]. 

### 2.2. Hematopoietic Stem Cell Transplantation (HSCT)

At the age of 12 years, the patient received a successful allogeneic HSCT, from an HLA-identical, unrelated female to correct the enzyme deficiency. The patient had full engraftment. Within 5 months after HSCT, the enzyme activity of the recipient’s lymphocytes increased to the normal range. No sign of acute or chronic GVHD was observed. For the successive 31 months, β-glucuronidase activity in her leukocytes rose to nearly normal levels, and the excretion of urinary GAGs was greatly diminished and normalized [8]. 

### 2.3. Clinical Course after HSCT

Clinical improvement, including motor function, was well recognized. The patient became ambulatory without aid and could even ride a bicycle and bathe alone. The recurrent infections of the upper respiratory tract and the middle ears decreased in frequency and severity, and dyspnea on exertion, snoring, and vertigo improved substantially [8]. At 12 years of age pre-HSCT, her intelligence quotient (IQ) was 50, i.e., the equivalent to the developmental level of a 6-year-old, but dropped from 50 to 47 two years after the HSCT [8] (Table 1).

At 29 years of age (17 years after HSCT), her IQ (Tanaka-Binet intelligence test V) dropped from 50 to 37 before and after HSCT (Table 1). She could walk 360 m alone in a 6 min-walk test. She has not suffered from upper respiratory infections for several years. She couldn’t calculate change for shopping by herself. She showed mild scoliosis, mild left ventricle hypertrophy, and moderate aortic valve regurgitation (Table 1). Ophthalmologic examination revealed no abnormalities in the cornea or retina. In the otolaryngological examination, tubing was inserted into the eardrum, and hearing tests showed moderate right-sided hearing loss and left-sided mixed hearing loss, respectively. The orthopedic assessment showed instability of the cervical spine and limited range of motion of the hip joint. The brain MRI showed no significant difference compared with that at pre-HSCT. β-glucuronidase activity in leukocytes was normal (Table 2). Excreted GAGs in urine were normal (Table 3). 

At 34 years of age (22 years after HSCT), her IQ (Tanaka-Binet intelligence test V) was 37, i.e., the same as at the age of 29. She can have a simple conversation but cannot calculate change for shopping. She retained her ambulatory mobility well. She sometimes experiences hip pain, but she can walk alone and climb upstairs using handrails. She may feel dizzy or lightheaded when tilting her head backward when looking up. Ophthalmologic examination revealed no abnormalities in the cornea or retina. In the otolaryngological examination, tubing was inserted into the eardrum. Hearing tests showed moderate right-sided hearing loss and left-sided mixed hearing loss, respectively; hearing loss on the left side was slightly lower than four years ago. Numbness and wobbling were observed during the rotation of the neck. In orthopedic assessment, the range of motion of the hip joint was limited but remained unchanged. Her respiratory function showed mild restricted impairment with a vital capacity (VC) of 69% and a forced expiratory volume/second (FEV1.0) of 1.5. Echocardiography showed moderate aortic regurgitation and mitral regurgitation, but cardiac function was not worsened compared with the findings at 29 years of age.

### 2.4. Radiological Findings

**Figure 1:**
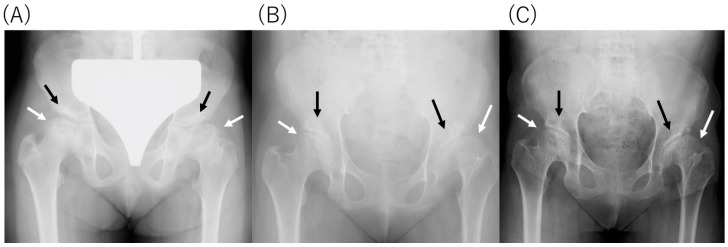
X-ray of the pelvis (**A**) pelvis at 9 years old, 3 years before HSCT, (**B**) pelvis at 15 years old, 3 years post-HSCT, (**C**) pelvis at 29 years old, 17 years post-HSCT. Dysplasia of the hip acetabulum (black arrow) and deformation of the femoral head (white arrow) are observed. No obvious narrowing of the joint space has been seen.

**Figure 2:**
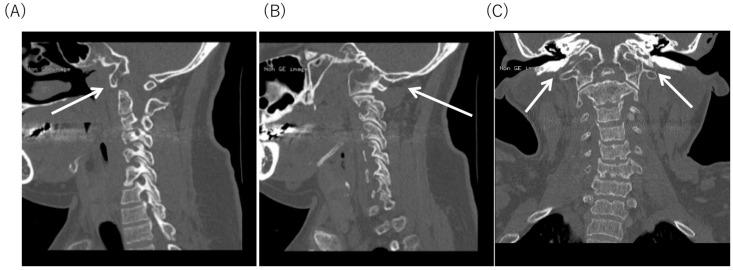
Cervical spine CT at 29 years old. Occipitalization of Atlas is seen. (**A**) Sagittal section. Occipitalization of the anterior arch of the first cervical vertebra and the lower surface of the occipital bone at the part indicated by the white arrow. (**B**) sagittal section and (**C**) coronal section. Occipitalzation of the lateral mass of the first cervical vertebra and the lower surface of the occipital bone are fused at the area indicated by the white arrow.

**Figure 3:**
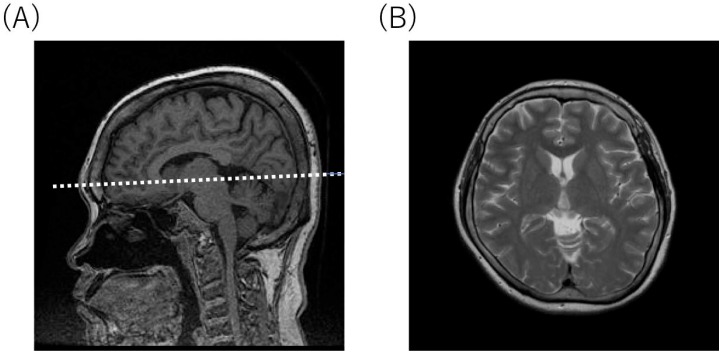
Brain MRI at 34 years of age (22 years post-HSCT). (**A**) Sagittal T1-weighted image. (**B**) Axial T2-weighted image. The white dotted line indicates the level of axial T2-weighted image shown in (**B**).

An X-ray of the hip revealed dysplasia of the hip acetabulum and deformation of the femoral head at age of 9, 15, and 29, respectively (Figure 1). Although there was no apparent progression with age, it was considered to be the cause of the limitation of the range of motion of the hip joint and hip pain. Cervical spine CT at 29 years old showed the occipitalization of atlas (also known as atlanto-occipital assimilation) that the first cervical vertebra was fused with the occipital bone at some sites (Figure 2). No obvious atlantoaxial subluxation was found. Brain MRI at the age of 34, i.e., 22 years after HSCT, showed no abnormal findings, e.g., atrophy of the cerebral cortex, ventricular enlargement, or cyst formation. (Figure 3).

## 3. Discussion

ERT is routinely and successfully used in patients with several types of MPS [7]. ERT using a recombinant human GUS enzyme, vestronidase alfa, was approved by the FDA and EMA in 2019. Regarding MPS VII, Fox et al. reported the use of the successful ERT in a 12-year-old boy with advanced MPS VII [6].

A pivotal phase-III trial on vestronidase alfa has shown a significant decrease in urinary CS-DS excretion after 24 weeks [5]. While a marked amelioration of organomegaly and fatigue was observed, only a relative improvement in the mobility and the respiratory functions was noted [5]. The recombinant enzyme is directly infused into the bloodstream allowing its delivery to peripheral organs except for the brain, because of its inability to cross the blood-brain barrier. To improve or prevent any potential neurological impairment, the application of HSCT should be considered. HSCT is based on the supply of deficient enzymes through nondeficient GUS-producing hematopoietic cells and its derivatives like brain microglia [10]. To date, 10 cases treated with HSCT have been reported in patients with MPS VII [3,8,11,12,13,14]; two of them underwent HSCT at a later stage, i.e., 7 and 12 years of age [3]. One patient was of unknown age, while the remaining seven underwent HSCT before 4 years of age [3,11,12,13,14]. Three of the nine patients died within a few years after HSCT [3,12]. Five of the seven surviving cases were reported within three years after HSCT [3,8,11,14]. It was thought that neurological development was often well maintained in patients who had no neurological symptoms at 3 years of age and who underwent HSCT before that time. In patients with neurological symptoms at 3 years of age, neurological development after HSCT was not improved but was maintained at the same or at a mildly reduced level. 

Two long-term follow-up reports after HSCT were reported [3,13]. The case of transplantation at 2 years of age and retransplantation at the age of three and a half years had scoliosis but proper neurological development for six years [13]. The case transplanted at 3 years of age showed moderate physical and neurological symptoms; however, the symptoms had been maintained for 12 years [3]. In this study, we have described the long-term course of the patient who received the first HSCT in MPS VII [8]. She underwent HSCT at the age of 12 years since she had moderate intellectual disability and difficulty walking, became wheelchair-bound, and had reduced motor skills. Two years after HSCT, she could walk again with the improvement of physical activity and ADL. Snoring was reduced, and respiratory function tests showed mild restrictive disorders which were maintained without deterioration. Her intellectual level decreased but was maintained for over 20 years post-HSCT. At the age of 12 years before HSCT, her IQ was 50, which was considered a developmental stage equivalent to that of a 6-year-old. Twenty-two years after HSCT, her IQ had dropped from 50 to 37. Although she did not show any improvement in development, she could live at a stage of development comparable to that of 6-year-old, with no loss of ability gained before and after HSCT. Twenty-two years after HSCT, changes in physical symptoms were observed due to the fusion of the first cervical vertebra and occipital bone, worsening of cervical vertebra instability, progression of degeneration of the hip joint, and mild deterioration of hearing. 

Tomatsu and coworkers described some genotype-phenotype correlations [15]. Our patient harbored the p.Ala619Val mutation associated with an attenuated phenotype [15]. The p.Ala619Val mutation is of great interest since it is specific to the Japanese population [15]. The p.Ala619Val mutation expressed 9.1% of normal cDNA in transfected COS cells. The residual activity of these expressed mutant proteins correlated with the attenuated phenotype for those mutations [15]. Cross-Sectional analysis showed that a higher residual β-glucuronidase enzyme activity in fibroblasts and lower GAGs excretion in urine were associated with longer survival [16]. Another Japanese patient with MPS VII with the p.ALa619Val mutation was diagnosed at the age of 24, but started walking at 2 years of age, had surgery for inguinal hernia at 4 years of age, and had mild gait disturbance [8]. At the age of 14, he had an IQ of 66 and a short stature, i.e., 125.2 cm (−5.5 SD). Until he was 30 years old, he was able to ride a bicycle and did not experience any deterioration in his athletic ability. In MPS VII, the correlation between genotype and phenotype has been reported [15], and p.Ala619Val is considered an attenuated type. From the comparison of these two cases, it was considered that among the attenuated types of p.Ala619Val, the symptoms varied among the patients. In this case, the improvement of motor function was observed by HSCT starting even at 12 years of age, although the slow progression of neurological findings might be due to the p.Ala619Val mutation.

Occipitalzation of the atlas is thought to be a manifestation of the so-called occipital vertebrae (that is, incomplete segmentation between the atlantal and occipital bone) [17]. Occipitalzation of the atlas is caused by the failure of the segmentation between the fourth occipital sclerotome (proatlas) and the first cervical sclerotome during embryonic development [18]. The incidence of occipitalization of the atlas ranges from 0.08% to 2.76% [19]. Occipitalization of the atlas and MPS VII may have been merged, but the careful follow-up was necessary due to increased instability of the cervical spine.

In conclusion, the current long-term observation post-HSCT in an MPS VII patient has demonstrated that HSCT has a therapeutic impact on motor function and ADL, and should be considered as a therapeutic option. 

It is critical that a well-trained medical team and facility provide multi-disciplinary care with the accompanying changes in skeletal dysplasia, hearing, visual acuity, cognitive function, ADL, respiratory function, sleep apnea, and *p* valvular heart disease.

To provide further improvement in the long-term prognoses of MPS VII patients, early diagnosis and treatment of ERT or HSCT are essential.

## Figures and Tables

**Table 1:** Clinical manifestations.

	5 Months before HSCT	15 Months after HSCT	17 Years after HSCT	21 Years after HSCT
age	12y2m	13y10m	29y11m	34y2m
Height (cm)	141.5 (−1.4 SD)	141.5 (−2.6 SD)	144.2 (−2.6 SD)	143.8 (−2.7 SD)
Weight (kg)	47.0 (+0.5 SD)	41.3 (−0.9 SD)	53.6 (+0.1 SD)	54.3 (+0.2 SD)
Body mass index	23.5	20.6	25.8	26.3
Intelligence quotient	50	47	37	37
Coarse facies	coarse	coarse	coarse	coarse
Corneal clouding	normal	normal	normal	normal
Thoracolumbar gibbus	abnormal	abnormal	abnormal	abnormal
Bone deformity	abnormal	abnormal	abnormal	abnormal
Hepatosplenomegaly	none	none	none	none
Snoring	severe	diminished	diminished	diminished
Deafness	moderate	moderate	moderate	moderate
Heart disease	Moderate MR Moderate AR	Moderate MR Moderate AR	Moderate MR Moderate AR	Moderate MR Moderate AR
Walking	Wheelchair	Ambulatory	Ambulatory	Ambulatory

SD: standard deviation, MR: mitral valve regurgitation, AR: aortic valve regurgitation.

**Table 2:** β-glucuronidase activity in lymphocytes before and after HSCT.

	before HSCT	17 Years after HSCT	Control
β-glucuronidase (nmol/mg protein/h)	ND	144.5	116.4–240.4
Arylsulfatase (nmol/mg protein/h)	NT	132.4	109.0–217.2
β-hexosaminidase (nmol/mg protein/h)	1427	NT	562–882

ND: not detected, NT: not tested.

**Table 3:** Urinary GAGs excretion before and after HSCT.

	5 Months before HSCT	15 Months after HSCT	17 Years after HSCT
Urinary GAGs (mg/g creatinine)	44.0	18.0	12.1
Control	14.2 ± 6.8	11.7 ± 5.7	10.3 ± 2.0

## Abbreviations

HSCT	Hematopoietic stem cell transplantation
MPS VII	Mucopolysaccharidosis type VII (Sly syndrome)
ADL	Activity of living
GAGs	Glycosaminoglycans
DS	Dermatan sulfate
HS	Heparan sulfate
CS	Chondroitin sulfate
LSD	Lysosomal storage disorder
CNS	Central nervous system
GUS	Glucuronidase
rhGUS	Recombinant human glucuronidase
ERT	Enzyme replacement therapy
MRI	Magnetic resonance imaging
IQ	Intelligence quotient
SD	Standard deviation
MR	Mitral valve regurgitation
AR	Aortic valve regurgitation
